# Reconstitution of human shelterin complexes reveals unexpected stoichiometry and dual pathways to enhance telomerase processivity

**DOI:** 10.1038/s41467-017-01313-w

**Published:** 2017-10-20

**Authors:** Ci Ji Lim, Arthur J. Zaug, Hee Jin Kim, Thomas R. Cech

**Affiliations:** 1 Howard Hughes Medical Institute, University of Colorado BioFrontiers Institute, Boulder, CO 80309 USA; 2 Department of Chemistry & Biochemistry, University of Colorado, Boulder, CO 80309 USA

## Abstract

The human shelterin proteins associate with telomeric DNA to confer telomere protection and length regulation. They are thought to form higher-order protein complexes for their functions, but studies of shelterin proteins have been mostly limited to pairs of proteins. Here we co-express various human shelterin proteins and find that they form defined multi-subunit complexes. A complex harboring both TRF2 and POT1 has the strongest binding affinity to telomeric DNA substrates comprised of double-stranded DNA with a 3′ single-stranded extension. TRF2 interacts with TIN2 with an unexpected 2:1 stoichiometry in the context of shelterin (RAP1_2_:TRF2_2_:TIN2_1_:TPP1_1_:POT1_1_). Tethering of TPP1 to the telomere either via TRF2–TIN2 or via POT1 gives equivalent enhancement of telomerase processivity. We also identify a peptide region from TPP1 that is both critical and sufficient for TIN2 interaction. Our findings reveal new information about the architecture of human shelterin and how it performs its functions at telomeres.

## Abstract (web summary)

The human shelterin complex protects telomere ends from being recognized as damaged DNA sites and regulates telomere length in conjunction with telomerase. Here the authors establish the stoichiometries of human shelterin complexes of various compositions and show shelterin provides dual pathways to stimulate telomerase processivity.

## Introduction

The human shelterin proteins are essential to protect the telomere ends from being recognized as damaged DNA sites and also serve to regulate telomere length, in conjunction with telomerase^1^. The removal or knockdown of shelterin proteins—TRF1, TRF2, RAP1, TIN2, TPP1, and POT1—can result in chromosome end fusions, leading to genome instability^2^, or failure to regulate telomerase recruitment and telomere maintenance^3, 4^. Indeed, single amino-acid mutants or deletion variants of TPP1^5^ and TIN2^6^ fail to recruit telomerase in humans, leading to telomere shortening and dyskeratosis congenita.

The shelterin proteins are known to interact with each other, mostly through binary interaction studies, and have been proposed to form a higher-order multi-subunit protein complex (known as either shelterin or telosome)^1, 7, 8^. TRF1 and TRF2 are homodimers^9–11^ and have a C-terminal DNA-binding Myb domain that binds specifically to telomeric duplex YTAGGGTTR repeats^10, 12^. The TRF proteins serve as the foundation for shelterin assembly at telomeres. Both TRF proteins interact with TIN2, which serves to enhance TRF2 stability at telomeres^13, 14^. TPP1 is recruited to telomeres via TIN2^7, 15^. The TIN2–TPP1 interaction is suggested to be the foundation for forming higher-order shelterin complexes^7^. POT1, the chromosome end-capping protein^16^, has two oligonucleotide/oligosaccharide-binding (OB)-folds that bind single-stranded telomeric DNA, TTAGGGTTAG^17, 18^, but it depends on its interaction with TPP1 for recruitment to telomeres in cells^19, 20^. Rap1 is recruited to telomeres via interaction with TRF2^21, 22^.

For shelterin protection of chromosome ends, TRF2 serves as the main antagonist to inappropriate recognition of DNA damage at telomeres by the ATM kinase pathway^1, 2, 23–25^, while POT1 blocks activation of the ATR kinase pathway at the single-stranded telomeric region^25^. Concerning shelterin recruitment of telomerase, TPP1 binds telomerase^26^ via the TPP1 TEL-patch^5^ and TERT (telomerase reverse transcriptase) TEN domain^27^. In addition to telomerase recruitment, TPP1 present in the TPP1–POT1 heterodimer has been shown to be a telomerase processivity factor in vitro^28^, and this is achieved by slowing telomerase dissociation from the DNA primer and aiding translocation^29^.

Even though we know the overall shelterin protein composition of telomeres^30^, we know very little about the stoichiometry of different possible variations of shelterin complexes, their protein–protein and protein–DNA stability, and also how they are assembled and distributed along telomeres. Knowing this information will contribute to understanding how these protein complexes perform their functions in telomerase recruitment, regulation, and telomere protection. Individual shelterin proteins have been successfully purified from both bacterial^21, 31^ and insect^17, 30^ cells and provided valuable information on their functions. Until very recently, the only binary shelterin complex studied in pure form has been a heterodimer consisting of POT1 bound to an N-terminal domain of TPP1^28^. Although human/mouse shelterin complexes immuno-purified from cell lysates have provided valuable insights regarding their composition, size, and DNA-binding properties^7, 8, 13, 32, 33^, the low yield and heterogeneity of partially purified protein complexes limit quantitative studies.

Here, we expressed and purified various human shelterin complexes in sufficient quantity for both biochemical and biophysical characterization. We focused on TRF2-containing shelterin complexes rather than those containing TRF1, given the profound importance of TRF2 for telomere structure^34^. We show that most of the shelterin complexes can be purified without dissociation, indicating they form stable complexes at least in vitro. The shelterin complex that has both TRF2 and POT1 binds to single-stranded/double-stranded telomeric DNA junctions preferentially to exclusively single-stranded or double-stranded telomeric DNAs. We measured the stoichiometry of several shelterin complexes assemblies and revealed TRF2 and TIN2 have an unequal 2:1 (TRF2:TIN2) stoichiometry. We also identified a peptide region from TPP1 that is critical and sufficient to interact with TIN2—a key interaction for shelterin complex assembly. We further show TPP1 tethered to a telomere via TRF2–TIN2 or via POT1 is sufficient to confer its telomerase processivity function.

## Results

### Reconstituted human shelterin forms stable complexes

**Fig. 1:**
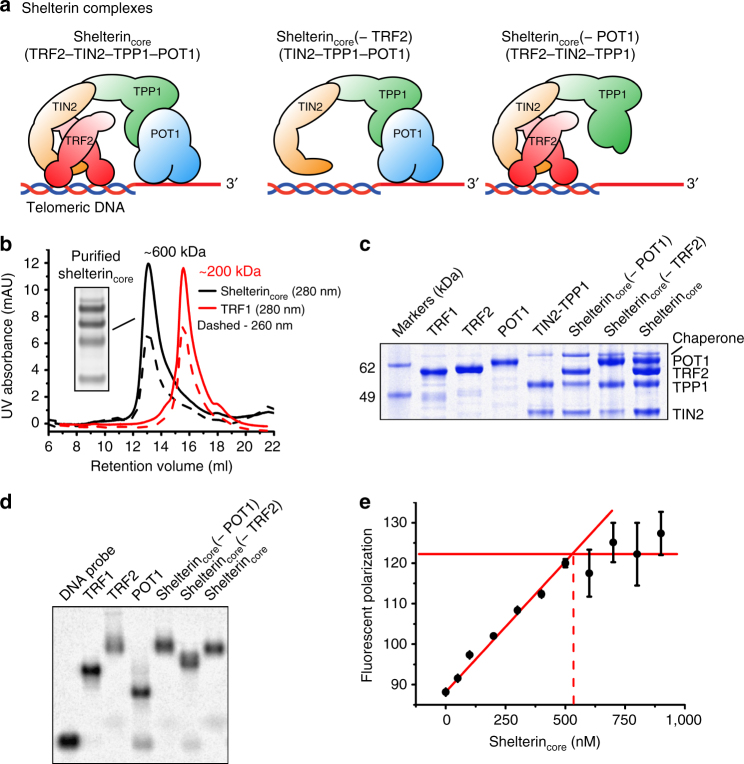
Human shelterin proteins form stable protein and DNA–protein complexes in vitro. **a** Cartoon schematics of shelterin complexes reconstituted in this work. **b** Representative size-exclusion chromatography trace of purified shelterin_core_ complex using Superose 6 10/300 increase column. The trace of TRF1 is included for comparison. **c** SDS-PAGE of purified recombinant human shelterin complexes expressed and reconstituted in *Tni* insect cells. Soluble purified individual shelterin subunits TRF1, TRF2, and POT1 are shown for comparison. The band above POT1 was identified via mass spectrometry to be the HSP70 chaperone. SDS-PAGE (sodium dodecyl sulfate-polyacrylamide gel electrophoresis) is visualized with Coomassie blue stain. **d** Agarose-EMSA shows stable protein–DNA complexes formed by recombinant human shelterin complexes containing TRF2, POT1, or both with ss/ds telomeric DNA substrate (C8). The telomeric ss/ds DNA substrate is ^32^P-5′-labeled (longer strand) and incubated with 500 nM protein for 30 min at room temperature before running on a 0.5% agarose gel. The experiments in **b**–**d** were repeated twice (independent experiments) to ensure consistency. **e** Saturation-binding assay to determine shelterin_core_ complex DNA-binding activity. 500 nM Alexa-488-5′-labeled C8 substrate was used in a fluorescent polarization assay with increasing shelterin_core_ concentration. Red lines are drawn for ease of visualization. (Error bars indicate range of data, *n* = 2 independent experiments.)

To express the various shelterin protein subunits and complexes, we used the insect cell/baculovirus system (*Tni* insect cells), which provides better machinery for folding mammalian proteins than bacterial systems. We co-expressed TRF2, TIN2, TPP1, and POT1 by simultaneous infection of cells with individual shelterin baculoviruses. We named the shelterin complex consisting of the four subunits (TRF2, TIN2, TPP1, and POT1) as the shelterin_core_ complex, defined as the minimal shelterin complex capable of binding both single-stranded and double-stranded telomeric DNA (Fig. 1a). Shelterin complexes consisting of TRF2–TIN2–TPP1 and TIN2–TPP1–POT1 are named shelterin_core_(−POT1) and shelterin_core_(−TRF2), respectively, with reference to their loss of single-stranded or double-stranded telomeric-binding properties. All shelterin proteins and complexes were purified with a final size-exclusion chromatography (SEC) step and a single-peak corresponding to the protein/complex was then collected to ensure protein/complex homogeneity.

All four shelterin proteins—TRF2, TIN2, TPP1, and POT1—elute in the same SEC peak (Fig. 1b), indicating the four proteins can form a stable shelterin_core_ complex. Shelterin_core_(−POT1) and shelterin_core_ (−TRF2) complexes are also stable as reconstituted complexes expressed in insect cells. Figure 1c shows the SDS-PAGE analysis of all purified shelterin proteins and stable complexes. To test the telomeric DNA binding of the purified shelterin protein complexes, we used a DNA substrate (annotated as C8) comprising both single-stranded and double-stranded telomeric regions separated by 8 bp of random double-stranded DNA to ensure the strand hybridization register. All tested shelterin proteins and complexes bound the C8 DNA substrate in an Agarose-electrophoretic mobility shift assay (Ag-EMSA) (Fig. 1d). Other than TRF2 (with some upwards smearing), all shelterin protein–DNA complexes migrated into the gel as single bands.

We then quantified the DNA-binding activity of the purified shelterin_core_ complex by performing a saturation-binding assay using fluorescence polarization. The C8 probe longer strand was 5′-labeled with Alexa-488 dye and its fluorescence polarization was measured with increasing shelterin_core_ complex concentration (Fig. 1e). The fluorescence polarization signal (500 nM DNA) increased linearly with shelterin_core_ concentration before leveling off at ~540 nM onwards, indicating the shelterin_core_:DNA stoichiometry is 1:1 and the protein complex is ~90% active for DNA binding.

### Shelterin_core_ binds best to ss/ds telomeric DNA junction

In order for the TPP1–POT1 complex or POT1 to localize to telomere ends in vivo, the train of TRF2–TIN2–TPP1–POT1 is required^35, 36^. We tested if such an interaction could be recapitulated in vitro with the shelterin_core_ complex. More specifically, would the combination of TRF2 and POT1 binding to double-stranded and single-stranded telomeric DNA, respectively, enhance shelterin complex affinity at telomere ends?

**Fig. 2:**
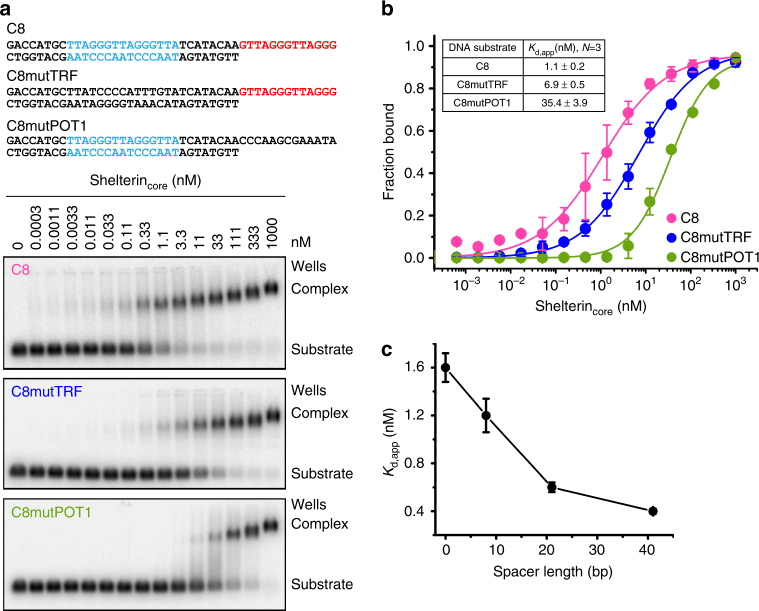
Shelterin_core_ complex binds strongest to telomeric DNA with both single-stranded and double-stranded regions. **a** Representative Ag-EMSA shows shelterin_core_ complex binding to a telomeric DNA substrate (C8) with binding sites for both TRF2 dimer and POT1 and to C8mutTRF and C8mutPOT1, which have mutated TRF2 dimer and POT1-binding sites, respectively. **b** The equilibrium dissociation constants (*K*_d,app_) were determined through curve fitting (with Hill equation) from the Ag-EMSA gels. Error bars represent s.d. (*n* = 3 independent experiments). **c** Binding affinity increases some what with increasing spacer length (base pairs) between TRF2 dimer and POT1-binding sites. (Error bars are s.d., *n* = 3 independent experiments.)

To delineate the contributions of TRF2 and POT1 DNA-binding in the context of a protein complex, we mutated the TRF2 and POT1-binding sites of the C8 DNA substrate to generate C8mutTRF (does not bind TRF2) and C8mutPOT1 (does not bind POT1) substrates. Figure 2a shows representative Ag-EMSA gels of the three DNA substrates with increasing shelterin_core_ complex concentration. C8 has an equilibrium dissociation constant (*K*_d_) of 1.1 ± 0.2 nM (Fig. 2b). C8mutTRF, which has a mutated TRF2-binding site, has *K*_d_ = 6.9 ± 0.5 nM while C8mutPOT1, which has a mutated POT1-binding site, has *K*_d_ = 35.4 ± 3.9 nM. Removal of POT1 from shelterin_core_ reduced its affinity for the C8 DNA by ~14-fold, and removal of TRF2 had an even larger effect (Supplementary Fig. 1). The binding of shelterin complexes to purely single-stranded or double-stranded telomeric DNA was also measured and, as expected, complexes containing POT1 bound telomeric ssDNA and those containing TRF2 bound telomeric dsDNA (Supplementary Fig. 2). All interactions showed substantial specificity for telomeric DNA sequence, although binding to non-telomeric DNA could be observed in the micro-molar concentration range (Supplementary Fig. 2). As a cautionary note, initial unoptimized protein preparations of shelterin_core_(−POT1) showed strong binding to telomeric single-stranded DNA which disappeared when the expression protocol was optimized. Overall, these experiments revealed the contributions of both TRF2 and POT1 to DNA-binding by the shelterin complex and sub-complexes.

To determine if the distance between the TRF2 and POT1-binding sites affects the shelterin_core_ complex affinity, we measured the binding to telomeric ss/ds substrates in which these sites were separated by spacers of 0, 8, 21, and 41 bp. We found that all of these DNA substrates formed high-affinity complexes; the shelterin_core_ complex *K*_d_ progressively became stronger with increasing spacer length, with 41 bp spacer having a *K*_d_ of ~0.5 nM (Fig. 2c). Taken together, we demonstrate that the shelterin_core_ complex binds telomeric substrates with both single-stranded and double-stranded regions (i.e., ss/ds telomere junction) more strongly than substrates with just either single-stranded or double-stranded telomeric DNA.

### Pre-bound shelterin_core_ prefers 3′ TTAGG telomeric ssDNA

**Fig. 3:**
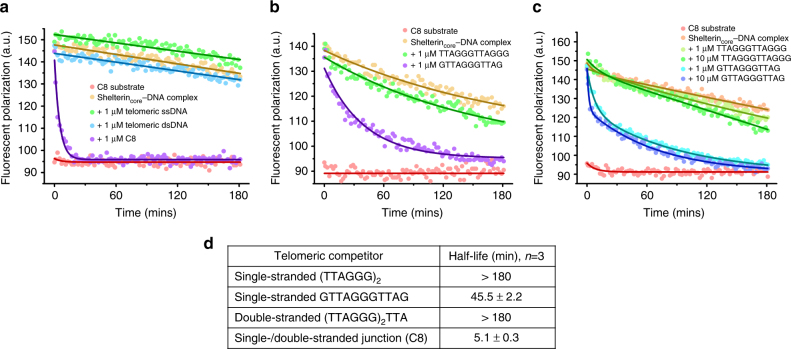
Shelterin_core_ complex bound at ss/ds telomeric junction remains stable in the presence of excess telomeric single-stranded or double-stranded DNA. **a** Fluorescence polarization assay to measure the dissociation rate of pre-formed shelterin_core_-Alexa488-C8 probe protein–DNA complex in the presence of excess single-stranded, double-stranded and ss/ds junction telomeric competitors. 5 nM of Alexa488-C8 probe was incubated with 100 nM of shelterin_core_ complex for 30 min before adding 1 µM competitor (200-fold excess to fluorescent probe). **b** Comparison of the complex dissociation rate with single-stranded telomeric competitors with either 3′ TTAGGG or TTAG. **c** Comparison of dissociation rates of complexes with 1 and 10 µM single-stranded telomeric DNA competitor. **d** Summary of complex dissociation rates in the presence of various telomeric competitors obtained from single-exponential decay function fitting. (Error bars are s.d., *n* = 3 independent experiments using 2 different protein batches.)

The human double-stranded telomeric region is on the order of several kb^37^ and thus it is in excess of the single-stranded region which is only several hundred nt^38^. This could potentially lead to sequestration of shelterin complexes away from the 3′ telomeric overhangs and cause telomere dysfunction. To determine if excess telomeric DNA substrates can compete the shelterin_core_ complex from ss/ds telomeric DNA, we measured the stability of the shelterin_core_-C8 protein–DNA complex in the presence of excess single-stranded or double-stranded telomeric DNA using the fluorescence polarization assay. The shelterin_core_ complex dissociated rapidly with a half-life of 5.1 ± 0.3 min from the labeled C8 DNA substrate when competed with excess C8 substrate, while it remained relatively stable when competed with either excess single-stranded (TTAGGG)_2_ or double-stranded (TTAGGG)_2_TTA DNA substrates (Fig. 3a). This suggests that once the shelterin_core_ complex binds to the ss/ds telomeric DNA junction, it remains relatively stable against a large background of double-stranded telomeric DNA; this would effectively target TPP1 and POT1 to the chromosome 3′ ends.

The high affinity and stability of shelterin_core_ at ss/ds telomeric junction could also prevent TPP1–POT1 or POT1 from binding to the 3′ end of the single-stranded telomeric tail, which would be a deleterious outcome. We therefore tested the stability of the shelterin_core_–C8 complex in the presence of single-stranded telomeric DNA ending with TTAG, which is the preferred human telomere overhang sequence^39^. We saw shelterin_core_ complex was effectively competed off C8 with excess GTTAGGGTTAG substrate with a half-life of 45.5 ± 2.2 min (Fig. 3b). Increasing the single-stranded telomeric DNA competitor concentration 10-fold did not change the half-life significantly (Fig. 3c), indicating that the competitor DNA traps the protein upon dissociation rather than actively attacking the protein–DNA complex. A table summarizing the half-lives is shown in Fig. 3d. From the above, there is clearly a strong preference among various DNAs concerning their ability to compete the shelterin_core_ complex from a ss/ds telomeric DNA junction: ss/ds junction >TTAG-ending ssDNA >TTAGGG ss or ds DNA.

### Human shelterin_core_ has unequal 2:1 TRF2:TIN2 stoichiometry

**Fig. 4:**
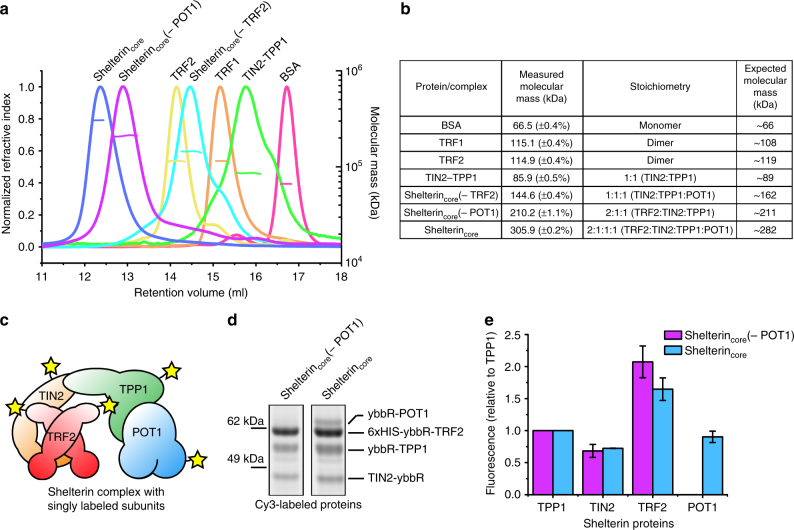
Human shelterin complexes have a 2:1 TRF2:TIN2 stoichiometry. **a** Size-exclusion chromatography multi-angle light scattering (SEC-MALS) assay to determine absolute molecular mass of various purified human shelterin complexes. **b** Summary of the shelterin complexes molecular mass (percentage error from software fitting) and the expected molecular stoichiometry. **c** Shelterin complexes were expressed with a single ybbR tag for each subunit. **d** The purified complexes were labeled with fluorescent dye (Cy3) for relative stoichiometry analysis in a SDS-PAGE gel by band fluorescence-intensity measurement. **e** The quantified fluorescence intensities were normalized to TPP1 counts and compared. (Error bars represent the range of data, *n* = 2 independent experiments using different protein batches.)

The stoichiometry of shelterin complexes is fundamental in building working models to understand how shelterin proteins are recruited and assembled at telomeres. The global stoichiometry of shelterin proteins at telomeres in cultured cells has been studied^30^ but does not address the stoichiometry of subunits within an individual shelterin complex. Here we used the SEC multi-angle light scattering (SEC-MALS) technique to measure the absolute mass of our purified shelterin proteins and complexes (Fig. 4a). The TRF1 measured mass is 115.1 kDa, which is closest to a predicted dimer mass of 108 kDa (Fig. 4b). Similarly, for TRF2, we obtained a mass of 114.9 kDa, which is closest to a dimer mass of 119 kDa. A dimeric state of TRF1 and TRF2 is consistent with previous literature^9, 11^. For TIN2–TPP1, we measured the mass to be 85.9 kDa, which is closest to a 1:1 heterodimer mass of 89 kDa. For shelterin_core_(−TRF2) complex, we measured the mass to be 144.6 kDa, which is closest to a 1:1:1 heterotrimer mass of 162 kDa.

Given that TRF2 is a dimer, one might expect each monomer to bind a TIN2 molecule, which would give a 2:2:2 stoichiometry of TRF2:TIN2:TPP1 or 2:2:2:2 stoichiometry of TRF2:TIN2:TPP1:POT1. However, by SEC-MALS the shelterin_core_(−POT1) and shelterin_core_ complex masses were 210.2 and 305.9 kDa, respectively. The closest stoichiometry match for shelterin_core_(−POT1) would be 2:1:1 (TRF2:TIN2:TPP1) which gives an expected mass of 211 kDa, while for shelterin_core_, the closest match is 2:1:1:1 (TRF2:TIN2:TPP1:POT1) which gives an expected mass of 282 kDa, or 2:2:1:1 which gives an expected mass of 321 kDa. To differentiate these two possibilities for shelterin_core_ complex stoichiometry and also to confirm shelterin_core_(−POT1) complex stoichiometry, we added an N-terminal ybbR tag^40^ to each shelterin protein. The tags did not interfere with complex assembly. We then fluorescently labeled the purified ybbR-tagged shelterin_core_(−POT1) and shelterin_core_ complexes, and quantified the fluorescence intensities from SDS-PAGE (Fig. 4c–e). The TRF2 band had almost twice as much fluorescent signal as the TIN2, TPP1, and POT1 bands (Fig. 4e). Thus, using two separate techniques, we show the TRF2:TIN2 stoichiometry to be an unequal 2:1 in the context of shelterin complexes. This is unexpected since it has been shown that a TRF2 monomer binds one TIN2 peptide (also shown vice versa)^14^ thereby predicting an equal stoichiometry of these subunits in the shelterin_core_ complex.

### TPP1_480–544_ is critical and sufficient for TIN2 interaction

**Fig. 5:**
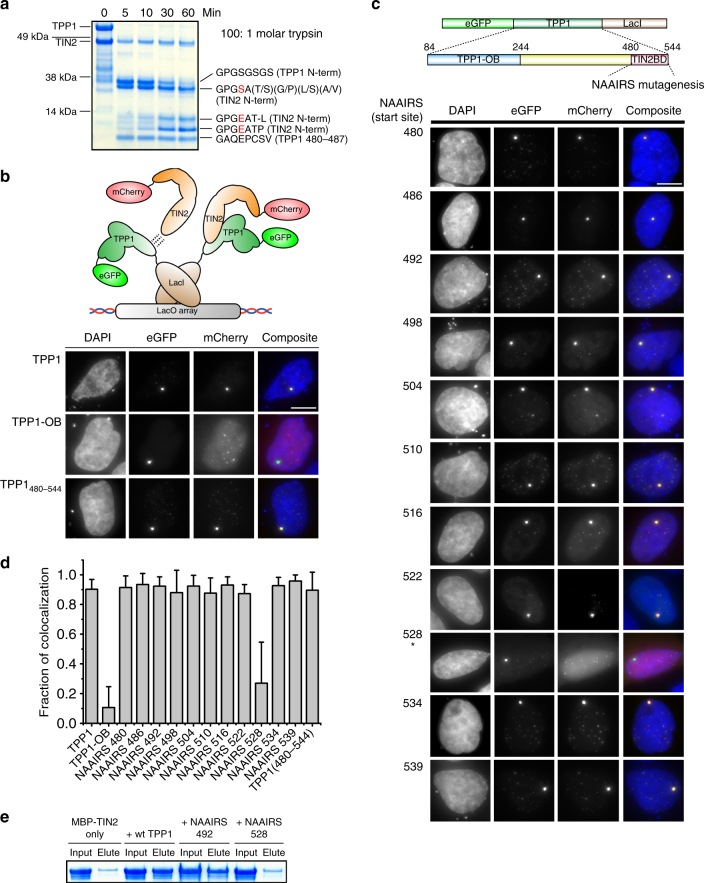
Identification of a TPP1 region critical and sufficient for TIN2 interaction. **a** Trypsin limited proteolysis of TIN2–TPP1 complex and peptide sequencing. Red letters in the peptide sequence denote mismatch to the wild-type TPP1 sequence. **b** Cartoon schematic showing the LacI–LacO array system used to test the TIN2–TPP1 interaction via fluorescence colocalization in U2-OS cells. Positive control shows full-length TPP1 (eGFP) co-localizing with TIN2 (mCherry). Negative control consisting of TPP1 OB-fold with the TIN2-interacting domain deleted (eGFP) shows multiple TIN2 spots (mCherry) that do not colocalize. Sufficiency for TPP1_(480–544)_ peptide to interact with TIN2 is also shown. Scale bar is 10 µm. **c** Top, schematic of TPP1 LacI construct region used for NAAIRS mutagenesis. Bottom, representative fluorescent images of nuclei from transfected cells. Asterisk points to the NAAIRS mutant that has the lowest fraction of colocalization (defined as colocalization of mCherry with a single GFP focus in a single nucleus). Scale bar is 10 µm. **d** Quantification of the fraction of colocalization between eGFP–TPP1 constructs and mCherry-TIN2 (Error bars are s.d., *n* > 50 nuclei per construct from 3 biological replicates). **e** Streptavidin-coated beads with biotinylated TPP1 480–544 peptide were used to capture MBP-TIN2. The amount of captured MBP-TIN2 is shown in the elute lanes. MBP-TIN2 captured by biotinylated TPP1 480–544 peptide with NAAIRS mutations at positions 492 (positive control) and 528 (affected region) are compared with the wild-type peptide. (Two independent experiments gave equivalent results.)

The TIN2–TPP1 interaction is known to be critical for higher-order shelterin complex assembly^7^. It is thus important to identify the regions of TIN2 and TPP1 that are involved in this interaction. Although deletion studies have identified regions of TIN2 and TPP1 that are critical for their interaction^7, 19, 41^, our purified complexes gave us the opportunity to elucidate a specific interaction patch on TPP1 and TIN2. The purified TIN2–TPP1 complex was subjected to limited proteolysis (Fig. 5a). N-terminal sequencing of the protected peptides revealed they were from the TIN2 N-terminus, the TPP1 N-terminus, and TPP1(480–487). MALDI further revealed the molecular mass of the TPP1(480–487) protected peptide to be 10.25 kDa, which indicates it to be TPP1_480–544_ including the 3× FLAG at the C-terminus. TPP1_480–544_ is 65 aa long and is consistent with having the C-terminal 22 aa that was previously identified to be critical for TIN2 interaction^19^. We did not obtain reliable mass for the other protected peptides from TIN2.

To identify region(s) within TPP1_480–544_ that are critical for TIN2 interaction, we used the LacI–LacO array system^26, 27^ to test TIN2–TPP1 interaction in cells upon TPP1_480–544_ NAAIRS mutagenesis^42^. The TPP1 domain was inserted between enhanced-GFP (eGFP) and LacI (Fig. 5b). The TPP1 constructs were co-expressed with mCherry-tagged TIN2 in the U2OS 2-6-3 cell line, which has a LacO array integrated in its chromosome 1^43^. Colocalization of the eGFP (green channel) and mCherry (red channel) at the single-LacO site would indicate interaction between TIN2 and TPP1. eGFP–TPP1–LacI was used as the positive control while eGFP–TPP1–OB–LacI was used as the negative control (Fig. 5b). TPP1 colocalized with TIN2 at 90% of the LacO sites while TPP1–OB, which does not interact with TIN2, colocalized at ~10% (Fig. 5d). We also tested the sufficiency of TPP1_(480–544)_ peptide to interact with TIN2 and showed ~90% colocalization between TPP1_(480–544)_ and TIN2 (Fig. 5b, d). All TPP1 NAAIRS mutants except TPP1_NAAIRS(528–533)_ colocalized with TIN2 at a level comparable to that of wild-type TPP1 (Fig. 5c). TPP1_NAAIRS(528–533)_ colocalized with TIN2 at only 25% of the LacO sites (Fig. 5d).

To further test if mutation at TPP1(528–533) disrupts the TIN2–TPP1 interaction, we performed a pull-down of maltose-binding protein (MBP)-TIN2 with resin-bound wild-type and mutant TPP1_480–544_ peptides. Wild-type and NAAIRS 492 peptides could pull-down MBP-TIN2 while NAAIRS 528 peptide pulled down substantially less MBP-TIN2 (Fig. 5e). In addition to confirming that the NAAIRS mutation at TPP1(528–533) can disrupt the TIN2–TPP1 interaction, these data also show that the TPP1_480–544_ peptide is sufficient for TIN2–TPP1 interaction.

### Tethering of TPP1 is sufficient for telomerase stimulation

**Fig. 6:**
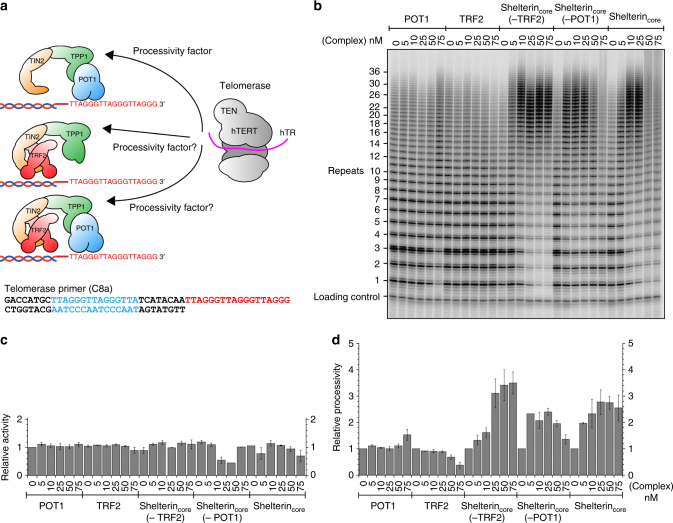
TPP1 tethering is sufficient for shelterin enhancement of telomerase processivity. **a** Cartoon schematics of the shelterin complexes tested and the primer used (C8a), which allows binding of TRF2, POT1 and telomerase. **b** Direct telomerase assay to determine the effects of shelterin complexes on telomerase activity and processivity. **c** Total activity of telomerase primer elongation products (normalized to a radioactive oligonucleotide loading control) with bound shelterin protein complexes at various concentrations (nM). **d** Telomerase processivity with each shelterin protein/complex. The processivity (relative to no-protein lane, which is normalized to 1) was calculated by dividing the counts >14 repeats by total counts. (Error bars in panels **c** and **d** indicate the range for two independent experiments.)

When TPP1–POT1 was identified as a telomerase processivity factor^28, 31^, TPP1 was recruited to the DNA substrate via its dimerization with POT1. However, POT1 is not required for telomerase recruitment to telomeres in vivo, where instead TIN2 recruits TPP1^15^. This suggests TPP1–POT1 may not be the major or only processivity factor for telomerase, and a shelterin complex such as shelterin_core_(−POT1) (TRF2–TIN2–TPP1), which can also potentially tether TPP1 to telomeres, may function as a more biologically relevant processivity factor for telomerase. To test this, we performed direct telomerase assays using shelterin_core_(−TRF2), shelterin_core_(−POT1) and shelterin_core_ complexes. These protein complexes were tested at increasing concentrations to ensure complete binding to the DNA. The telomerase primer used, C8a, contains a double-stranded telomeric region for TRF2 binding and a single-stranded region for telomerase and POT1 binding (Fig. 6a). In vivo reconstituted human core telomerase (hTERT and hTR) was immuno-purified from HEK293T cells.

Addition of POT1 or TRF2 alone to the telomerase extension reaction had only small effects on telomerase activity and processivity (Fig. 6b–d), consistent with previous studies^28, 44, 45^. The shelterin_core_(−TRF2) complex (includes TPP1–POT1) increased the processivity of human telomerase ~3-fold (Fig. 6d), which is consistent with a previous TPP1–POT1 study^5^. Similarly, shelterin_core_(−POT1) and shelterin_core_ also increased the telomerase product size distribution (Fig. 6b). Both shelterin_core_(−POT1) and shelterin_core_ increased the telomerase processivity ~2–3-fold (Fig. 6d). Total telomerase activity did not change with the addition of shelterin proteins/complexes (Fig. 6c). In addition, in the absence of a TRF2-binding site, shelterin_core_(−POT1) stimulated processivity only to a small extent (Supplementary Figs. 3 and 4) similar to that of TPP1-N alone^28^, indicating TRF2 ds telomeric DNA-binding to be a major factor for the observed telomerase stimulation by shelterin_core_(−POT1). Overall, the above results demonstrate that the TPP1–POT1 heterodimer complex is not exclusive in stimulating telomerase processivity, and shelterin_core_(−POT1) can serve the same function with equivalent efficiency in vitro.

### RAP1 inclusion does not affect shelterin_core_ functions

**Fig. 7:**
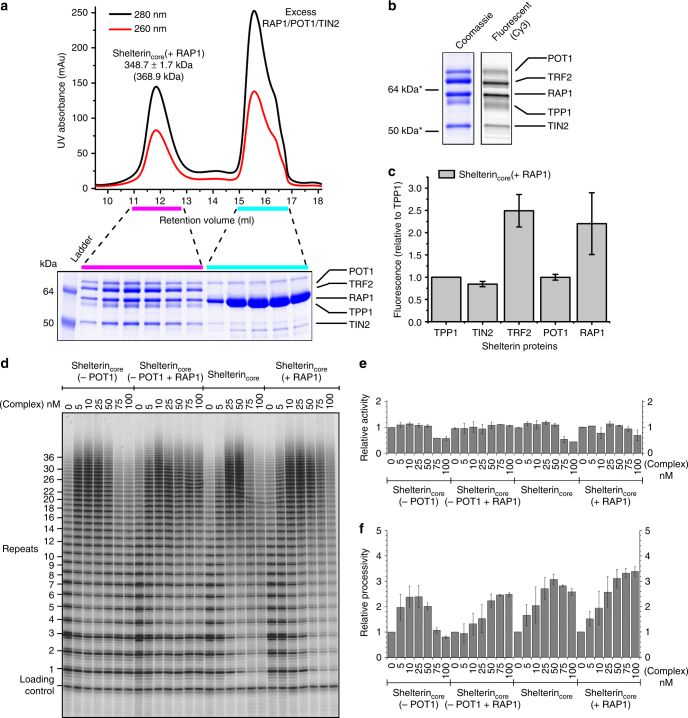
Incorporation of RAP1 into shelterin_core_ complex does not change TRF2:TIN2 complex stoichiometry, and there are two each of RAP1 and TRF2. Telomerase stimulation by shelterin complexes is also not affected by RAP1 inclusion. **a** Superose 6 gel-filtration trace of co-expressed shelterin_core_ (+RAP1) complex (RAP1–TRF2–TIN2–TPP1–POT1) showed a mono-disperse peak (left peak) with SDS-PAGE showing five distinct Coomassie-stained bands representing the five shelterin proteins. SEC-MALS analysis determined the protein complex to be 348.7 ± 1.7 kDa, with the bottom number in parentheses being the theoretical mass for a 2:2:1:1:1 (RAP1:TRF2:TIN2:TPP1:POT1) complex. The right peak is composed of excess un-incorporated RAP1, POT1 and TIN2. **b** Purified singly ybbR-tagged shelterin_core_ (+RAP1) complex labeled with Cy3-CoA dye and compared with Coomassie-stained bands as reference. *Molecular weight is an apparent value of SeeBlue Plus2 protein marker (Thermo Fisher) on a tris-glycine gel. **c** Quantification of fluorescence-intensity showed TRF2:TIN2 relative stoichiometry to be 2:1 (*P* < 0.05) while TRF2:RAP1 is 1:1. Error bars indicate s.d. for three independent experiments performed with three different protein preparations. **d** Direct telomerase assay to determine the effects of RAP1 inclusion on processivity stimulation by shelterin_core_(−POT1) or shelterin_core_ complexes. **e** Total activity of telomerase primer elongation products (relative to no-protein lane) with bound shelterin protein complexes at various concentrations (nM). **f** Telomerase processivity (relative to no-protein lane) with each shelterin protein/complex. (For panels **e** and **f**, error bars indicate the range for two independent experiments.)

In vivo, TRF2 is complexed with RAP1^46, 47^, and this complex is important in protecting telomeres from inappropriate homology-directed repair^48^. Having determined the shelterin_core_ complex stoichiometry, a major question is if incorporation of RAP1 into the complex changes the complex stoichiometry of TRF2–TIN2, especially since RAP1 interacts at a TRF2 region near TRF2’s TIN2-binding site^22^. To answer this question, we purified the shelterin_core_ (+RAP1) complex (RAP1, TRF2, TIN2, TPP1, and POT1) to high homogeneity (Fig. 7a). SEC-MALS analysis determined the complex mass to be ~350 kDa, which is between 322.2 kDa (shelterin_core_ + 1 RAP1) and 368.9 kDa (shelterin_core_ + 2 RAP1) (Supplementary Fig. 5). To differentiate between these two possible stoichiometries, every subunit was singly tagged with ybbR (Fig. 7b). Quantification showed the 2:1 TRF2:TIN2 complex stoichiometry was not changed and TRF2:RAP1 had a 1:1 (2:2 in a complex) stoichiometry (Fig. 7c). In addition, using SEC-MALS, we found that our preparations of RAP1 alone behaved as a monomer in solution (Supplementary Fig. 5), contrary to the tetramer reported previously^49^.

In terms of DNA interactions, we found shelterin_core_ (−POT1 + RAP1) complex had no significant changes in DNA-binding preferences as compared to shelterin_core_ (−POT1) (Supplementary Fig. 6a). Similar results were obtained for shelterin_core_ (+RAP1) as compared with shelterin_core_ (Supplementary Fig. 6b), indicating RAP1 incorporation in human shelterin complexes has no significant impact on TRF2 and POT1 DNA-binding properties. Next, we asked if telomerase stimulation by RAP1-less shelterin complexes are affected by RAP1 inclusion. We found that shelterin_core_ (−POT1 + RAP1) and shelterin_core_ (+RAP1) still stimulate telomerase processivity (Fig. 7d). Quantification confirmed that RAP1 inclusion did not affect telomerase activity (Fig. 7e). Maximal processivity stimulation was the same for all four complexes (~2.5–3 fold) although the RAP1 complexes were able to sustain processivity stimulation at higher protein concentrations more effectively than complexes without RAP1 (Fig. 7f). Overall, we show RAP1 incorporation has no effect on human shelterin_core_ complex stoichiometry and has minimal effects on telomeric DNA-binding and telomerase stimulation.

## Discussion

Our finding that various forms of human shelterin can be expressed and purified as discrete protein complexes has provided much new biophysical information, such as the equilibrium binding constants of these complexes for telomeric ds and ss DNA substrates. This information is in general agreement with that recently reported for mouse shelterin containing the POT1a subunit^32^. In addition, our work has provided molecular-level insight regarding three biological questions about shelterin: (1) How can POT1–TPP1 find the telomere 3′ end, given that TRF-containing complexes bound on dsDNA might sequester the POT1–TPP1? (2) Are alternative ways of tethering TPP1 near the telomere 3′ end equivalent in providing enhanced telomerase processivity (3) How should cartoons of the human telomere be drawn, given that TRF1 and TRF2 are homodimers and could plausibly each bind two TIN2 subunits? These questions are addressed below.

The existence of human shelterin as a multi-protein complex is supported by studies in which shelterin complexes were immuno-purified from cultured cell extracts and the presence of shelterin proteins in the complex was identified by specific antibodies^7, 8^. Further characterization of these immuno-purified shelterin complexes was not feasible given the limited materials obtained. In this work, we successfully expressed and purified various human shelterin protein complexes to high homogeneity and quantity. We show the human shelterin proteins can stably exist as several protein complexes including TRF2–TIN2–TPP1 (shelterin_core_(−POT1)), TIN2–TPP1–POT1 (shelterin_core_(−TRF2)), and TRF2–TIN2–TPP1–POT1 (shelterin_core_).

The double-stranded region of a telomere is several kb long^37^, potentially providing a vast excess of binding sites to sequester the shelterin_core_ complex away from the few hundred nt single-stranded region. As shown here, the dual-binding mode (ss and ds DNA-binding) property of the shelterin_core_ complex helps overcome this problem. More specifically, a shelterin complex harboring both TRF2 and POT1 (shelterin_core_ complex) binds telomeric ss/ds junctions with higher affinity than purely ss or ds telomeric regions, similar to the mouse shelterin complex^32^. This also suggests that shelterin complexes without POT1 will most likely populate the double-stranded telomeric region. Since POT1 is about ~5–10-fold sub-stoichiometric relative to TRF1 and TRF2^30^, most of the TRF1 and TRF2-harboring shelterin complexes are unlikely to have POT1 and would bind only to telomeric dsDNA, which is a less stable interaction. This can explain why in live cells, the majority of the TRF1 and TRF2 were highly dynamic, while a fraction of TRF2 had the same slower dynamics as POT1^50^. It is interesting to note, unlike TRF2, TRF1 did not have a slower dynamic fraction, and this may be explained by the poor connectivity between TRF1 and the rest of shelterin as determined by mass spectrometry^47^.

**Fig. 8:**
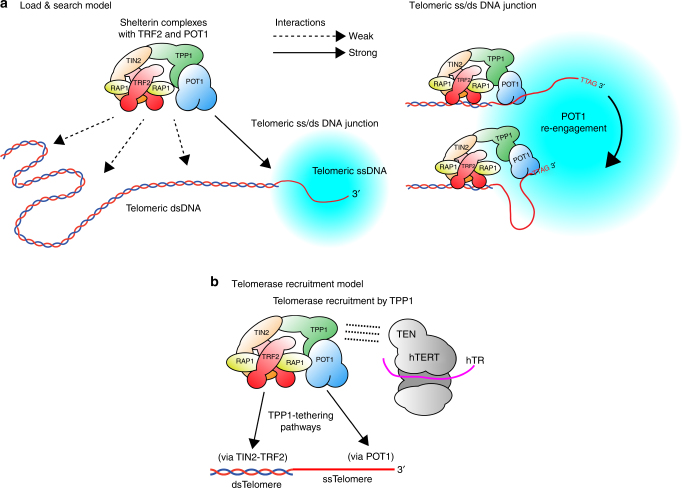
Models for shelterin search mechanism for telomeric 3′ end and telomerase recruitment based on the shelterin_core_ complex. **a** A model for shelterin positioning at the telomeric 3′ end suggests a shelterin complex such as shelterin_(core)_ would first localize to a ss/ds telomeric DNA junction via TRF2 and POT1 binding (shown here via internal ss-telomeric region). Then the POT1 component would seek the higher affinity 3′ TTAG end, thus protecting the chromosome end. **b** Dual pathways exist for TPP1 tethering to telomeres, either via TIN2–TRF2 or via POT1, both of which would facilitate telomerase recruitment and its processivity

Because shelterin complexes having both TRF2 and POT1 will likely populate the ss/ds telomeric junction regions, this will effectively position TPP1–POT1—a key functional element of shelterin telomerase regulation-nearer to the 3′ ends. We also showed that once shelterin_core_ complex binds to a ss/ds telomeric substrate, it is unlikely to move to a single-stranded telomeric DNA. At first glance, this seems like it could potentially sequester TPP1–POT1 from finding the very 3′ ends, but one possible model to reconcile the above facts is a “load & search” model (Fig. 8a). For example, a shelterin_core_ complex first searches for telomeric ss/dsDNA through a three-dimensional diffusion mechanism^32^ (probably through multiple re-engagements) and binds a ss/ds telomeric DNA junction, with POT1 binding to a nearby telomeric ssDNA region (e.g., an internal telomeric ssDNA site). From there, POT1 scans along the telomeric ssDNA until it finds the 3′ end and is stabilized there due to its higher affinity to the 3′ terminal sequence TTAG^39^. Eventually, the 3′ end of the chromosome will be looped back, forming a loop that is bridged by shelterin proteins, unlike a T-loop that is formed by strand-invasion^51^. The model is consistent with the suggestion that TPP1–POT1 can slide along single-stranded DNA^52^. Our off-rate data also showed single-stranded telomeric DNA ending with TTAG is a better competitor than DNA ending with TTAGGG, further supporting the model that the POT1 in the shelterin_core_ complex will seek higher affinity sites. Because higher-order shelterin complexes such as shelterin_core_ have a higher propensity to find chromosome 3′ ends, their TPP1 subunit will also serve to recruit telomerase to the 3′ end. Similarly, it will also deliver POT1 to the single-stranded region to inhibit activation of the ATR kinase pathway^25^. Therefore, determining which shelterin sub-complexes exist at telomeres and how they are distributed along telomeres in vivo will be essential in furthering our understanding of shelterin functions.

The TPP1–POT1 heterodimer is sufficient to enhance telomerase processivity in vitro^28^. TPP1 provides the TEL patch that directly interacts with telomerase^5, 26, 53^, while POT1 brings the TPP1 to telomeric ssDNA^29^. However, in vivo, it appears that recruitment happens the other way around—TPP1 recruits POT1^19, 20^—and it is TIN2 that brings TPP1 to the telomere^15, 36^. Here we resolve this potentially confusing situation by showing that tethering of TPP1 to the DNA substrate is sufficient for it to be a telomerase processivity factor. This tethering can be accomplished by TIN2–TRF2 or by POT1 (Fig. 8b). Even though we did not include TRF1 in this study, based on our findings, it is reasonable to suggest a shelterin complex such as TRF1–TIN2–TPP1 can be a telomerase processivity factor as well.

Previously POT1–TPP1 was shown to give a 2–3-fold increase in telomerase processivity^5^. These measurements were done with a truncated complex comprised of TPP1-N (amino acids 87–334) plus POT1. Here, we find that the POT1 with the entire TPP1 protein plus the TIN2 subunit increases telomerase processivity about three-fold. Thus, these additional protein domains do not have a substantial effect on stimulation of telomerase processivity. In summary, our study documents a new avenue to achieve telomerase recruitment and processivity by shelterin proteins (Fig. 8b). This pathway, which had not been previously shown in vitro, depends on TPP1 tethering via TRF2–TIN2 but is independent of POT1. This may have relevance to the situation in vivo, where POT1 is not required for shelterin–telomerase interaction^15^. It remains to be determined which pathway telomerase uses for recruitment to telomeres in cells.

In this work, we measured the stoichiometry of a human shelterin complex containing full-length TRF2, RAP1, TIN2, TPP1, and POT1. Interestingly, the TRF2:TIN2 stoichiometry in the context of the protein complex is 2:1 (i.e., one homodimer of TRF2 per TIN2). The unequal 2:1 stoichiometry differs from a previous structural study of the TRF1, TRF2, and TIN2 interaction, which suggested a 1:1 stoichiometry instead^14^. However, the difference could be due to the previous study using peptides instead of full-length proteins as used in this work. Because the TIN2-interacting domain of TRF2 maps in the TRF2 homodimerization domain^14^, it is possible that the conformation of this region of TRF2 only allows a single TIN2 to dock. Furthermore, secondary structure prediction shows that the TIN2 N-terminus has several blocks of alpha helices, which would be more rigid than unstructured peptide and perhaps give steric hindrance, preventing two proteins from docking. An untested assumption is that the stoichiometry of shelterin overexpressed in insect cells predicts the stoichiometry of the natural shelterin complex residing on human chromosome telomeres.

In terms of potential biological implications, the uneven stoichiometry of TRF2–TIN2 effectively reduces the number of TIN2–TPP1–POT1 recruited to telomeres. This can be a mechanism for cells to limit the number of TPP1–POT1 at telomeres and thus directly affects DNA protection and telomerase regulation functions of shelterin. It will be interesting to determine if the TRF1–TIN2 stoichiometry is the same as TRF2–TIN2 and if the TRF1–TIN2–TRF2 trimeric complex has a different stoichiometry due to potential steric hindrance when TIN2 binds both TRF1 and TRF2.

We also identified a TPP1 peptide that can recruit TIN2 independently. Secondary structure prediction shows two 10 amino-acid alpha helices, of which one overlaps with the region TPP1_528–533_ where our NAAIRS mutation affects TIN2 interaction. Further work will be required to elucidate which residue(s) are critical for TIN2 interaction. Since the affinities of TRF1 and TRF2 to the TIN2 peptide have been measured to be ~0.3 and 6.5 µM, respectively^14^, it will be interesting to determine the binding affinity of TPP1_480–544_ to TIN2 and compare the protein–protein affinities. In addition, TPP1–POT1 is sub-stoichiometric relative to TIN2 and TRF2 in cells^30^, making understanding how TPP1 interacts with TIN2 critical for studies of shelterin telomere biology.

Because detailed measurements were not available, published cartoons of the human shelterin complex show different versions of TRF2:TIN2 stoichiometry-one TRF2 per TIN2^1, 54^ or two TRF2 per TIN2^3, 55^. Different cartoons also show one or two RAP1 per TRF2 dimer^56–58^. Based on the stoichiometry of human shelterin expressed in insect cells, pending verification of the stoichiometry of the complex in vivo, we now suggest that these cartoons should be drawn with one TIN2 molecule and two RAP1 molecules per TRF2 dimer. The addition of RAP1 did not affect the TRF2:TIN2 stoichiometry, which remained 2:1.

## Methods

### Protein expression

Human TRF1 (NP_059523.2), TRF2 (XP_005256180.1), RAP1 (NP_061848.2), TIN2 (NP_036593.2), TPP1 (AAX82621.1), and POT1 (NP_056265.2) cDNAs were individually cloned into the pFastBac1 expression vector (Invitrogen). The TRF1, TRF2, and RAP1 constructs have N-terminal hexa-histidine (6XHis) tags while TIN2, TPP1, and POT1 have PreScission-cleavable N-terminal 6XHis-MBP tags. The expression vectors were used to make recombinant baculoviruses based on the protocol established in the Bac-to-Bac Baculovirus Expression System (Invitrogen). The baculovirus stocks were titered by gp64 detection using flow cytometry analysis (Expression Systems). Yield-optimized amounts of baculoviruses were used to infect 2–4 liters of *Tni* cells (Expression Systems) at a cell density of 1.5–2.0 × 10^6^ cells ml^−1^. The infected cells were then incubated in a shaker for 66–72 h (27 °C, 130 rpm) before they were collected. The collected cells were either immediately used for protein purification or snap-frozen using liquid nitrogen for later purification.

### Purification of shelterin protein complexes

All steps were performed at 4°C. Cell pellets were disrupted in lysis buffer (50 mM HEPES pH 7.5, 300 mM NaCl, 1 mM DTT, 1% NP-40, 1 mM PMSF, EDTA-free protease inhibitors (Roche)) and the lysate was clarified by centrifugation. The supernatant was mixed with pre-equilibrated amylose beads for 4 h. The beads were washed three times with wash buffer (50 mM HEPES 7.5, 300 mM NaCl, 1 mM TCEP) before eluting the captured proteins with elution buffer (50 mM HEPES pH 7.5, 300 mM NaCl, 1 mM DTT, 10 mM maltose). The eluted proteins were then concentrated to ~10 mg ml^−1^ before cutting with PreScission protease (GE Healthcare Life Sciences) overnight. The MBP tag cleavage efficiency was checked with SDS-PAGE before exchanging the buffer to low-salt buffer (50 mM HEPES pH 7.5, 150 mM NaCl, 1 mM DTT) for anion-exchange chromatography (HiTrap Q HP, 5 ml) with AKTA explorer FPLC machine (GE Lifesciences). After injecting the sample into the column, the column was washed with 3× C.V. of low-salt buffer before eluting with a linear buffer gradient change to 60% high-salt buffer (50 mM HEPES pH 7.5, 1000 mM NaCl, 1 mM DTT) over 10 C.V. Cleaved MBP tags were removed during the column wash and the tag-free protein complexes were collected during elution phase. SDS-PAGE was used to determine which elution fractions to pool and concentrate. The samples were then injected into a Superose 6 increase 10/300 GL column pre-equilibrated with storage buffer (50 mM HEPES pH 7.5, 300 mM NaCl, 1 mM TCEP) for the final SEC purification step. The elution fractions were checked with SDS-PAGE before pooling and concentration. The concentrated proteins (>5 mg ml^−1^) were then aliquoted and snap-frozen before storage at −80 °C.

### Agarose-electrophoretic mobility shift assay

Radioactive (5′-^32^P) DNA substrates (sense-strand labeled) were used for detection. The estimated specific activity is at least 200,000 c.p.m.per pmol. A reaction volume of 10 µl was used for the protein–DNA interaction with final 1000 c.p.m. radioactive substrate. The binding buffer was 50 mM Tris-HCl pH 8.0, 150 mM KCl, 2 mM MgCl_2_, 1 mM DTT, 10% (v/v) glycerol, 0.5 mg ml^−1^ BSA, and 0.02 mg ml^−1^ non-specific carrier dsDNA (5′-GACCATGCTTATCCCCATTTGTATCATACAA-3′). The reaction was incubated for 30 min at 23 °C before loading onto a 10 cm 15-well 0.7% agarose gel (SeaKem LE Agarose, Lonza). The electrophoresis buffer was 1× TBE and the gel was run for 1.5 h at 7 V cm^−1^ and 4 °C. The gels were then vacuum-dried for 1.5 h at 80 °C before exposure to phosphor screens. After overnight exposure, the screens were imaged with a Typhoon trio scanner (GE Lifesciences). The analysis of EMSA densitometry was done with Fiji (Distribution of ImageJ)^60^ and data fitted with the Hill equation using OriginPro (OriginLab). Independent replicates (Number of replicates, *n*, is stated in legend for each corresponding figure) were done and the average was fitted. The sequences for single-stranded DNA, ss-TEL is 5′-TTAGGGTTAGGGTTAGGG-3′, ss-nonTEL is 5′-GACCATGCTTATCCCCATTTGTATCATACAA-3′ while double-stranded DNA, ds-TEL is 5′-GACCATGCTTAGGGTTAGGGTTATCATACAA-3′, and ds-nonTEL is 5′-GACCATGCTTATCCCCATTTGTATCATACAA-3′.

### Fluorescence polarization

The C8 substrate sense-strand oligo was synthesized with a 5′ Alexa-488 fluorescent dye (5′-Alex488N-GACCATGCTTAGGGTTAGGGTTATCATACAAGTTAGGGTTAGGG-3′) and thermally annealed with the anti-sense strand (5′-TTGTATGATAACCCTAACCCTAAGCATGGTC-3′) (Integrated DNA Technologies). Each 40 µl reaction (buffer—50 mM Tris-HCl pH 8.0, 150 mM KCl, 2 mM MgCl_2_, 1 mM DTT, 10% (v/v) glycerol, 0.5 mg ml^−1^ BSA) contained 5 nM (500 nM for saturation-binding assay) fluorescent substrate and was incubated with protein for 30 min at 23 °C before fluorescent polarization measurement in a 384-well plate with Synergy 2 multi-mode plate reader (BioTek). For competitor assays, initial measurements were taken (defined as *t* = 0) before adding competitors and taking measurements every 2 min for 4 h.

### SEC-MALS

All protein samples were filtered with a 0.1 µm filter and adjusted to 1 mg ml^−1^ before injecting (100 µl sample volume) into a pre-equilibrated GE Superose 6 increase 10/300 GL column that was connected to a Wyatt Dawn EOS 18-angle light-scattering detector and Optilab DSP refractive index detector for multi-angle light scattering analysis. The SEC-MALS runs were conducted at 0.5 ml min^−1^ and 25 °C. The buffer used was 50 mM HEPES pH 7.5, 300 mM NaCl and 1 mM TCEP. The SEC-MALS analysis was done using Astra software.

### ybbR-tagged protein CoA-dyes labeling

ybbR tag of amino-acid sequence DSLEFIASKLA was added to the N-termini of TRF2, TPP1, and POT1 and C-terminus of TIN2 in the baculovirus constructs. Expression and purification were done similarly as described in the protein expression and purification sections. The purified protein complexes (5 µM) were then labeled with CoA-Cy3 (10 µM) using recombinant Sfp phosphopantetheinyl transferase (1 µM) in final buffer condition of 50 mM HEPES pH 7.5, 300 mM KCl, 10 mM MgCl_2_, 1 mM TCEP. After overnight incubation at 4 °C, the labeled protein complex was subjected to SDS-PAGE and the fluorescent gel was scanned with a Typhoon Trio Scanner.

### Limited proteolysis of protein complex

Trypsin (Promega) was used for limited proteolysis at 100:1 (protein:trypsin) molar ratio. Trypsin was added to 100 µl of 10 µM TIN2–TPP1 complex solution with final trypsin concentration of 0.1 µM. Digested samples were taken out at 0, 5, 10, 30, and 60 min and reactions quenched by formic acid addition. The samples were then run on SDS-PAGE to analyze digested protein bands. SDS-PAGE gels were blotted onto a polyvinylidene difluoride (PVDF) membrane and stained with Coomassie Blue for visualization. The transferred bands were excised and sent for Edman sequencing analysis (UC Davis Proteomics Core Facility, USA).

### Cell culture

*Tni* cells (Expression Systems) were cultured as suspension cells at 27 °C and 130 rpm in ESF 291 medium (Expression Systems) supplemented with a final 5 µg ml^−1^ gentamicin (Sigma-Aldrich). The cell density was maintained at 0.5–3.0 × 10^6^ cells ml^−1^. The U2-OS cells (2-6-3 cell line, a kind gift from David Spector) were cultured as adherent cells at 37 °C, 5% CO_2_ in DMEM medium supplemented with 2 mM l-glutamine, 1% penicillin/streptomycin, 100 µg ml^−1^ hygromycin B (for LacO array maintenance), and 10% fetal bovine serum.

### LacI–LacO array interaction assay

eGFP–TPP1–LacI and mCherry-TIN2S-expressing plasmids were co-transfected into the U2-OS cells containing the LacO array (grown on 24-well glass plates) using jetPRIME (Polyplus transfection) transfection reagent per manufacturer’s protocol. Twenty-four hours after transfection, the cells were washed twice with PBS and then fixed with 0.4% (v/v) paraformaldehyde in PBS for 5 min at room temperature. The cells were permeabilized for 5 min with 0.01% Triton-X 100 in PBS and finally stained with DAPI. The fixed cells were than imaged for DAPI, GFP and mCherry fluorescence. Colocalization analysis was performed with JACoP^60^ (a ImageJ plugin).

### Biotinylated peptide pull-down assay

For the purification of recombinant MBP-TIN2, the full sequence DNA was amplified from human cDNA by PCR and cloned into the *E. coli* expression vector pET28 (Novagen). The resulting MBP-TIN2 was expressed by IPTG induction (0.1 mM, 18 °C overnight) in *E. coli* Rosetta 2(DE3) (Novagen) and purified with amylose affinity chromatography followed by FPLC (previously described). For the peptide pull-down assay, peptides-wt TPP1, NAAIRS 492 and NAAIRS 528 mutants based on TPP1_480–544_ (GAQEPCSV…GSEPTPM)-were synthesized as C-terminal biotinylated peptides (Zhongping Tan & Xiaoyang Guan, CU Boulder, USA). The biotinylated peptides (10 µM) were incubated with MBP-TIN2 (10 µM) in storage buffer for 30 min at RT before mixing with streptavidin beads (Thermofisher Scientific) for another 30 min. The beads were washed 3× with storage buffer to remove uncaptured complexes before eluting by boiling at 95 °C for 5 min. The input and elute samples were subjected to SDS-PAGE analysis.

### Telomerase purification and telomerase direct assay

Plasmids expressing hTR and hTERT, a gift from J. Lingner (EPFL, Lausanne, Switzerland), were transiently transfected into HEK 293T cells with Lipofectamine 2000 reagent (Invitrogen). The over-expressed human telomerase was purified as previously described^59^. Shelterin protein complexes varied between 0 and 100 nM as indicated in figures. Double-stranded oligonucleotide substrate C8a was at 1 or 10 nM. Conditions for assays were 50 mM Tris-HCl pH 8.0, 50 mM KCl (15 mM brought in with telomerase, 35 mM added), 30 mM NaCl (brought in with shelterin complexes), 2 mM MgCl_2_, 1 mM spermidine, 5 mM μ-mercaptoethanol, 0.33 μM ^32^P-dGTP (3000 Ci mmol^−1^), 2.9 μM cold dGTP, 0.5 mM dATP, and 0.5 mM TTP.

Prior to starting telomerase extension, the protein complexes, human telomerase and substrate oligo were incubated at RT for 30 min. To start reaction, 3 μl of dNTP mix was added. Tubes were placed at 30 °C for 1 h. The reaction was stopped by the addition of 100 μl 3.6 M NH_4_Ac containing 20 μg glycogen, and 3000 c.p.m. of each of three oligonucleotide loading controls, LC1, LC2, and LC3. Following the addition of 500 μl of ethanol, samples were placed at −80°C overnight. Products were pelleted at 15 K r.p.m. for 15 min at 4 °C. After washing the pellet with 1000 μl of −20 °C 70% ethanol, the pellet was dried and then dissolved in 11 μl water plus 11 μl Sample prep buffer (0.1× TBE, 93% Formamide, 50 mM EDTA, 0.05% Bromophenol blue, and 0.05% Xylene cyanol). An aliquot of 10 μl of each sample was loaded on a 10% acrylamide 7 M urea sequencing gel, pre-run for 45 min at 90 W (constant). The gels were run for 1 h 45 min (short run) or 2 h 45 min (long run) at 90 W. Gels were dried and exposed to a phosphor screen before scanning.

### Data Availability

The data that support the findings of this study are available from the corresponding author upon reasonable request.

## Electronic supplementary material

Supplementary Info
