# Supplementary material for: Reconstitution of human shelterin complexes reveals unexpected stoichiometry and dual pathways to enhance telomerase processivity
Source: Nat Commun. 2017 Oct 20;8:1075. doi: 10.1038/s41467-017-01313-w (PMC5651854; doi:10.1038/s41467-017-01313-w)
Supplement: Supplementary file 1 — Supplementary Info [file 41467_2017_1313_MOESM1_ESM.pdf]

Supplementary Materials

Supplementary Figures

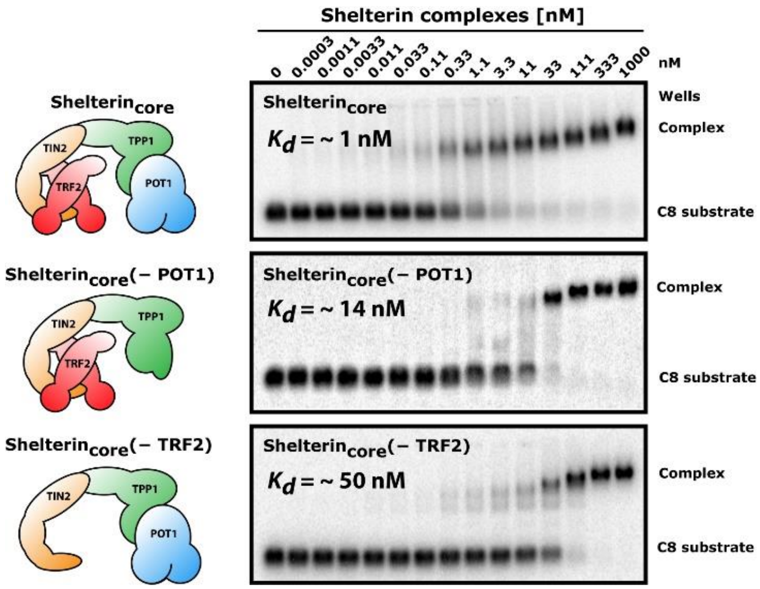

Supplementary Figure 1 Shelterin$_{core}$ complex and its sub-complexes bind to the C8 DNA substrate, which has both single- and double-stranded telomeric DNA sequences. Shelterin$_{core}$ has the highest affinity, followed by shelterin$_{core}$(− POT1) and shelterin$_{core}$(− TRF2). The indicated K_d values were obtained from three independent experiments using ≥ two protein preparations.

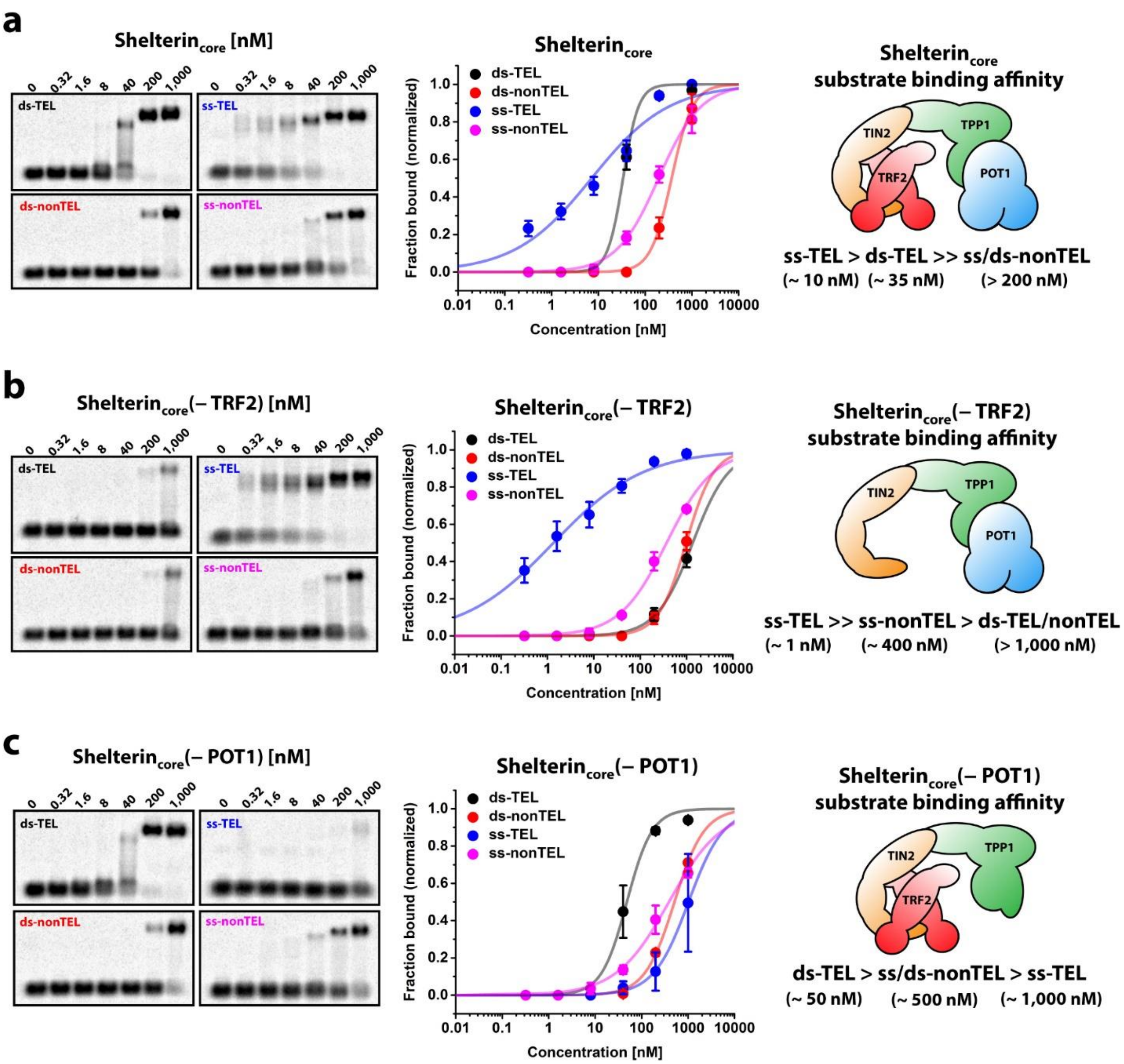

Supplementary Figure 2 DNA-binding affinities of Shelterin$_{core}$ complex and its sub-complexes to single- or double-stranded DNA (non-telomeric vs. telomeric). **(a)** Shelterin$_{core}$ complex has the highest affinity to single-stranded telomeric DNA (ss-TEL), followed by double-stranded telomeric DNA (ds-TEL) and has the lowest affinity to single- or double-stranded non-telomeric DNA (ss-nonTEL or ds-nonTEL). The binding curves are shown beside the representative gel images, followed by the complex cartoon with order of DNA substrate preferences. **(b)** Shelterin$_{core}$ complex (− TRF2) complex has the highest affinity to ss-TEL, followed by ssDNA and then ds-TEL/nonTEL. **(c)** Shelterin$_{core}$ complex (− POT1) complex has the highest affinity to ds-TEL, followed by ss-nonTEL/ds-nonTEL and then ss-TEL. (Error bars are s.d., *n*=3 independent experiments using ≥ 2 protein preparation batches.)

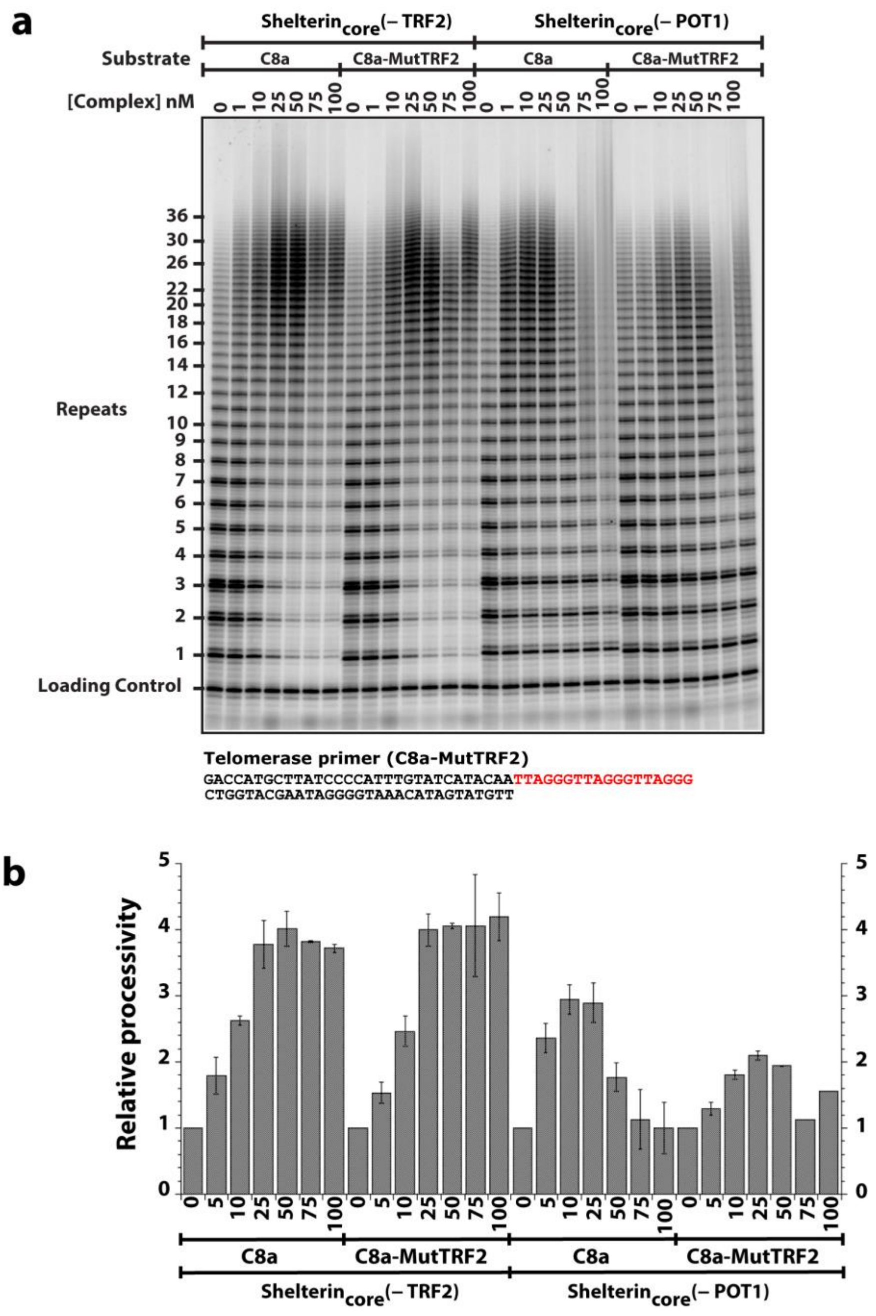

Supplementary Figure 3 Direct telomerase assay with shelterin$_{core}$(− TRF2) and shelterin$_{core}$(− POT1) complexes using C8a primer with mutated TRF2-binding site. **(a)** Direct telomerase assay using 10 nM C8a or C8a-MutTRF2 substrate which does not have TRF2-binding site. **(b)** Quantification of the relative processivity (calculated by dividing the counts > 14 repeats by total counts and relative to normalized “no protein” lane). Error bars show the range of two independent experiments.

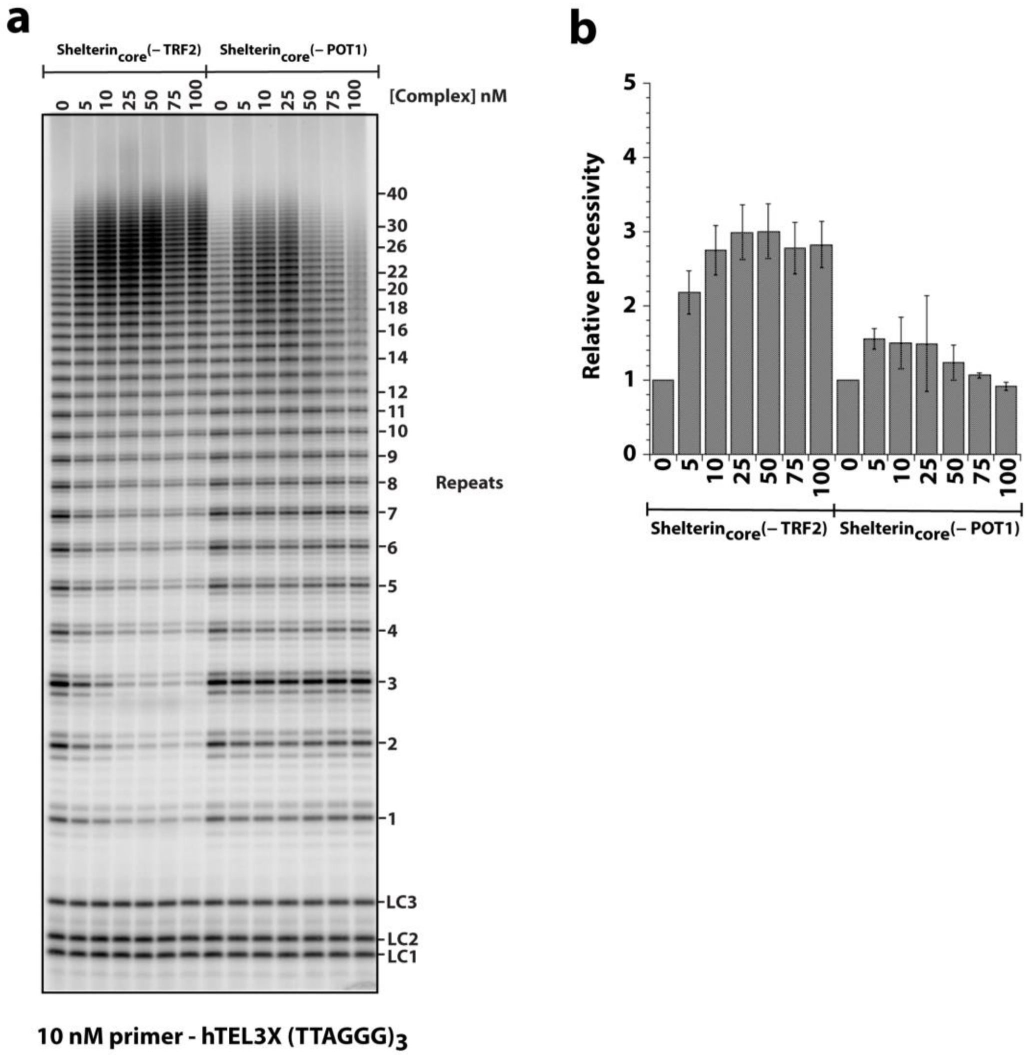

Supplementary Figure 4 Direct telomerase assay of shelterin$_{core}$(− TRF2) and shelterin$_{core}$(− POT1) complexes using single-stranded primer substrate, $(TTAGGG)_3$. **(a)** Direct telomerase assay using 10 nM single-stranded primer $(TTAGGG)_3$ which does not have a TRF2-binding site. **(b)** Quantification of the relative processivity (calculated by dividing the counts > 14 repeats by total counts and relative to normalized "no protein" lane). Error bars are s.d. with *n* = 5 independent experiments.

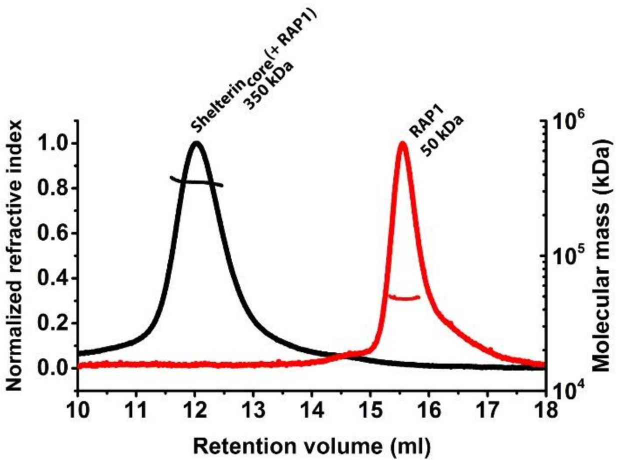

Supplementary Figure 5 Size-exclusion chromatography – multi-angle light scattering (SEC-MALS) measurement of shelterin$_{core}$(+ RAP1) complex and RAP1 alone. Superose 6 gel-filtration column was used for the sizing before MALS detection. The measured molecular mass of each protein/complex is indicated below the peak's label.

a

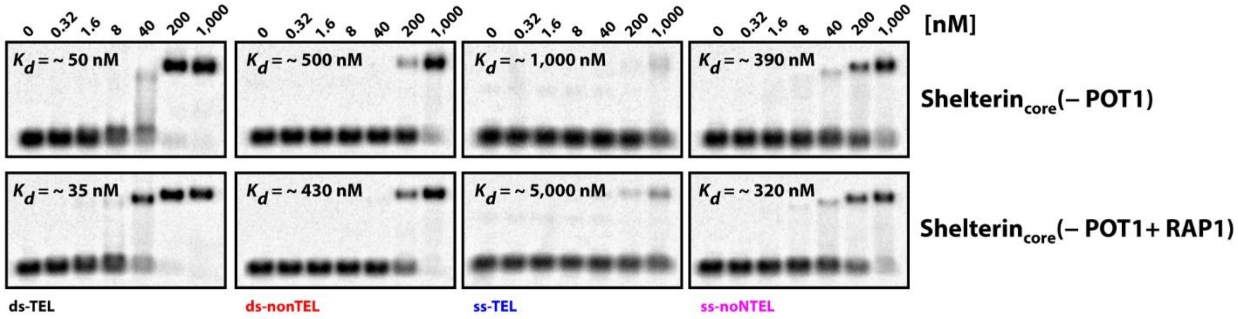

b

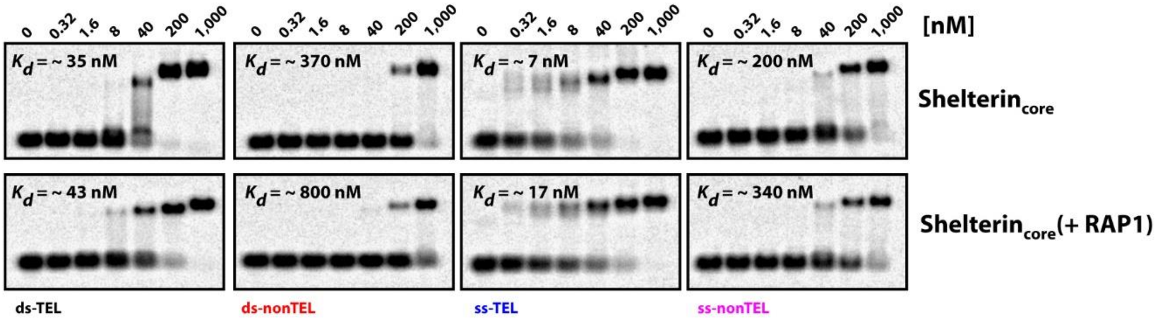

Supplementary Figure 6 Minimal effects of RAP1 incorporation on shelterin$_{core}$(− POT1) and shelterin$_{core}$ DNA-binding properties. **(a)** Binding of shelterin$_{core}$(− POT1 + RAP1) complex (all subunits co-expressed in insect cells) as compared to shelterin$_{core}$(− POT1) to four different DNA substrates to show affinity and specificity. **(b)** Comparison of DNA binding for shelterin$_{core}$ and shelterin$_{core}$(+ RAP1) complexes. Indicated K_d values were obtained from three independent experiments.
